# Rewriting Cardiovascular Disease: Are MicroRNAs the Next Frontier?

**DOI:** 10.3126/nje.v16i1.92324

**Published:** 2026-04-02

**Authors:** Indrajit Banerjee, Jared Robinson, Shradha Banerjee, Doané Robinson, Indraneel Banerjee

**Affiliations:** 1 Sir Seewoosagur Ramgoolam Medical College, Belle Rive, Mauritius; 2 Joe Morolong Memorial Hospital, Vryburg, South Africa; 3 Royal College Port Louis, Mauritius; 4 University of Cape Town, South Africa; 5 University of Florida Health, Jacksonville, United States of America

## Background

MicroRNAs were discovered roughly two decades ago, and our understanding of their function and clinical implications has advanced immensely. As technology has improved and mankind has made strides forward in the understanding of molecular medicine, enhancing understanding of disease mechanisms at the genetic and cellular levels, enabling the development of more precise and targeted therapeutic approaches. MicroRNAs are a class of RNA (ribonucleic acid) about 20 to 22 nucleotides long that bind to mRNAs (messenger RNA). Their main action is seen at the posttranscriptional level, where they aid in the regulation of gene expression. Simply put, MicroRNAs act in two ways to ultimately prevent or stop a protein from being produced. 1) Enable and promote the degradation of the mRNA. 2) Inhibit the translation process of an mRNA into a protein. Ultimately MicroRNAs interfere with the mRNA step in protein synthesis and break the chain of protein production [1]. MicroRNAs are essentially double-edged in nature as their action is dependent on the specific genes which are targeted. MicroRNAs may cause a disease to progress if there is an overexpression, or an under expression of certain proteins. The ability to attenuate the protective effects of “good” proteins via the manipulation of MicroRNAs and or the suppression/under expression of “bad” proteins may be the future of medicine. Where pathological processes are stopped and or prevented at the molecular level. MicroRNAs have been found to be of clinical importance in a wide set of medical conditions ranging from Cancer, to Neurological disorders and Cardiovascular disease [2].

### MicroRNAs and their implication in Cardiovascular disease

Cardiovascular disease is the leading cause of mortality worldwide, claiming the lives of roughly 18 million individuals annually. The most commonly implicated types of cardiovascular disease are heart failure, hypertension and its related complications, CVA (cerebrovascular accident)/ strokes, and CAD (coronary artery disease)/ heart attacks [3]. MicroRNAs have been found to have a highly active role in the pathogenesis and progression of Cardiovascular disease. The top 10 studied MicroRNAs in cardiovascular disease are namely miR-1, miR-208, miR-21, miR-29, miR-126, miR-145, miR-133, miR-155, miR-30 and miR-499 [4-13].

**miR-1**

has the physiological role of regulating the movement of ions across channels in the cardiac myocytes (the building block of cardiac tissue) which help to maintain a sinus cardiac rhythm, it can however become dysregulated and lead to an arrhythmia [4].

**miR-208**

is involved in the modulation of the myosin genes and thereby aids in the control of cardiac myocyte expression. If overexpressed or upregulated they lead to cardiac hypertrophy which is enlargement of the heart muscle and this ultimately ends in form of cardiac failure [5].

**miR-21**

regulates extracellular matrix deposition via the activation of fibroblasts and is physiologically used to repair tissues after they sustain an injury and or insult. If dysregulated or overexpressed miR-21 leads to cardiac fibrosis [6].

**miR-29**

is a regulator of genes which act on both the extracellular matrix and collagen and its physiological function is to decrease or suppress cardiac fibrosis, pathologically a reduced amount of miR-29 or if under expression occurs, the fibrosis in not kept in balance and excessive fibrosis of the cardiac muscle will occur [7].

**miR-126**

is involved in the endothelium where it aids in cell survival and the migration of cells. At physiological levels miR-126 aids the process of angiogenesis as well as the growth of new endothelium in the repair of the cardiac vasculature, if under expressed it has been found to play a vital role in the process of the development of atherosclerosis [8].

**miR-145**

has an action on the smooth muscles of blood vessels. It aids in the regulation of the differentiation of smooth muscle within the vascular walls. A reduction or decreased amount of miR-145 results in vascular disease via plaque formation [9].

**miR-133**

regulates genes which effect the growth of cardiomyocytes and in doing so aids in preventing cardiac hypertrophy. A reduction in the expression of MiR-133 results in fibrosis of the myocytes as well as cardiac hypertrophy [10].

**miR-155**

is involved in immune and inflammation via signaling pathways, and thereby regulates immune responses. In higher levels it leads to increased inflammation of the vessels and ultimately atherosclerosis [11].

**miR-30**

is a regulator of cell apoptosis and has a protective function in cardio-myocytes. Abnormal levels or functioning of miR-30 will lead to cardiac failure and premature cell death [12].

**miR-499**

has a more diagnostic function in the sense that is raised in acute myocardial infarctions and is said to have a protective effect on the cardiac muscle [13].

### The therapeutic and clinical implications

The potential and the development of the use of MicroRNAs will open up a new frontier in molecular cardiology and will further expand the frontier of precision medicine. MicroRNAs are almost limitless in their capabilities therapeutically, as they can be mimicked or inhibited, depending on their specific mechanism. For example, anti-miRs or antagomirs can be implemented when a specific MicroRNAs causes disease progression. The use of these antagomirs will thus disrupt or reduce the negative effects of the pro-disease action of that specific MicroRNA. The inhibition of miR-21 is of therapeutic importance. As described above, miR-21 regulates extracellular matrix deposition via the activation of fibroblasts and is physiologically used to repair tissues after they sustain an injury and or insult, essentially it promotes cardiac fibrosis. The inhibition of miR-21 will result in decreased cardiac fibrosis and thereby ensure an improved cardiac function. Another such example is miR-92a, which physiologically inhibits angiogenesis; the inhibition or blockage of this mechanism will result in increased angiogenesis, which will be useful in repairing and protecting cardiac tissues, ensuring they are well perfused post a myocardial infarction [6,14].

Mimics of MicroRNAs can be implemented when the specific MicroRNA is protective in nature, but just limited in its supply. The specific MicroRNA can be replaced and thus enhance its protective effects. miR-126 is involved in the endothelium where it aids in cell survival and the migration of cells. At physiological levels miR-126 aids the process of angiogenesis as well as the growth of new endothelium in the repair of the cardiac vasculature, if under expressed it has been found to play a vital role in the process of the development of atherosclerosis. Therefore, the supplementation and or replacement of the miR-126 will aid in improving vascular healing [8,15].

The clinical implication for microRNAs is at the cutting edge of molecular medicine, MicroRNAs are optimal biomarkers due to their stable levels within the bloodstream and the ease at which they can quantified within the blood. For example, MiR-208 is a MicroRNA which is highly cardiac specific in nature and can potentially be used as an early marker for myocardial infarctions, furthermore the use of such MicroRNAs can be extrapolated to aid in the prognosis of a patient’s condition as well as risk stratification. The use of MicroRNAs as biomarkers can also aid in tapering and guiding of therapies and drug selection in cases of heart failure. The potential of MicroRNAs as biomarkers in cardiovascular health spans across multiple pathological conditions including myocardial infarctions, arrhythmias, cardiac failure and even atherosclerosis [16].

There are a host of challenges with the implementation of MicroRNAs in everyday therapy, drug delivery is one of the biggest challenges as MicroRNAs are required to have a specific action on a specific tissue, this is difficult to achieve when the MicroRNAs will be administered systemically. Certain biological barriers further compound this issue as the brain and heart may see a decreased penetration of the MicroRNA therapy and thereby a decreased action where they are most needed. The drug delivery systems are still in their infancy and are at this point in time not very cost effective, various mechanisms of drug delivery of being tested and developed such as the use of viral vectors and lipid nanoparticles [17].

Off-target effects and related toxicities are the primary challenges in MicroRNAs based therapeutics, arising from the intrinsic ability of each MicroRNA to regulate multiple gene target [18]. On 2018, ONPATTRO (patisiran) an small interfering RNA targeting transthyretin MicroRNA was approved by the US- FDA for the treatment of hereditary transthyretin amyloidosis [19].

## Conclusion

It is evident that MicroRNAs will be a vital tool that both saves a countless number of lives as well as aids in reducing the mortality and morbidity in patients suffering from chronic diseases, most specifically in cardiovascular diseases. Their clinical and therapeutic potential are almost limitless and will someday be a mainstay in patient management and treatment. Before they can be deployed on a global level, further research and technological advancement is required in order to overcome the challenges of drug delivery and cost.
